# Investigation of the effects of benznidazole on the salivary glands: A biochemical, morphological, and functional approach

**DOI:** 10.1371/journal.pone.0317876

**Published:** 2025-06-03

**Authors:** Emanuelly Camilly Soares de Lima-da-Silva, Wallacy Watson Pereira Melo, Paulo Fernando Santos Mendes, Cristian dos Santos Pereira, José Mario Matos-Sousa, Cristian Kallahan Silva Chagas, Hannah Gil de Farias Morais, Roseana de Almeida Freitas, Antonio Hernandes Chaves-Neto, Rafael Rodrigues Lima

**Affiliations:** 1 Laboratory of Functional and Structural Biology, Institute of Biological Sciences, Federal University of Pará, Belém, Pará, Brazil; 2 Department of Oral Pathology, Federal University of Rio Grande do Norte, Natal, Rio Grande do Norte, Brazil; 3 Department of Basic Sciences, School of Dentistry of Araçatuba, Universidade Estadual Paulista, Araçatuba, São Paulo, Brazil; Museu Paraense Emilio Goeldi, Brazil

## Abstract

This study aimed to evaluate the possible biochemical effects of the administration of benznidazole on the parotid and submandibular salivary glands and saliva of rats, as well as on salivary components for the first time. Male Wistar rats (*Rattus norvegicus*), 66-days-old, weighing approximately 250 g were randomized into two groups: control, administered distilled water by gavage and benznidazole, administered benznidazole at a dose of 19.6 mg/kg daily by gavage over 15 days. On the 16th day, the animals were anaesthetized and the parotid and submandibular salivary glands and saliva were collected for oxidative biochemistry and morphometric analyses, and the biochemical composition of the saliva was also assessed. On the 16th day, the animals were anesthetized and their pilocarpine-induced saliva was collected. They were then euthanized to collect the parotid and submandibular salivary glands for oxidative biochemical and morphometric analyses, and the biochemical composition of the saliva was also assessed. Shapiro–Wilk normality analysis and Student’s t-test was used for parametric data and non-parametric data, respectively, with a significance of p < 0.05. The oxidative biochemical results revealed a reduction in the antioxidant capacity of the parotid and submandibular glands in benznidazole group. Morphometric analyses revealed an increase in the average area of the acini in both glands and a reduction in the stromal area of the parotid gland in the benznidazole group. Salivary analyses demonstrated a decrease in antioxidant levels and an increase in pro-oxidant levels in the group exposed to benznidazole. Total proteins, amylase, and mucin levels reduced in the exposed group. Thus, administration of benznidazole conclusively caused biochemical alterations in the antioxidant and morphological capacities of the salivary glands, followed by biochemical and protein alterations in the saliva, highlighting the possible damage caused by the drug in patients during the treatment of Chagas disease.

## 1. Introduction

Chagas disease (CD), widely known as American trypanosomiasis, is an infectious parasitic disease caused by the protozoan *Trypanosoma cruzi*, which is classified as a neglected disease that mainly affects underdeveloped or developing countries [1] and still represents a growing problem for public health worldwide [2]. Recently, CD has affected approximately eight million people and is responsible for the mortality of a large proportion of the population. Moreover, approximately six million people have been infected with CD, 28 million are at risk of infection by the disease, with an annual mortality rate of more than 10 thousand people [3].

In this context, the two medications primarily used to treat CD are benznidazole (BNZ) and nifurtimox, with only BNZ being sold in some countries, such as Brazil, USA and Europe [4,5]. BNZ is the first-choice medication for CD treatment because of its performance and biopharmaceutical effects with better therapeutic response and cure rate [6]. In addition, BNZ can treat acute and chronic phase CD [4], with reservations in the case of chronic patients with severe heart disease [7].

BNZ is a pro-drug, whose mechanism of action has not yet been completely elucidated. The drug undergoes a reduction process because of the action of a type II nitroreductase within the cell, causing the formation of free radicals and nucleophilic metabolites [8,9]. The reaction resultant nitro radical causes disruption of the fundamental macromolecules of the parasite, such as DNA (both mitochondrial and nuclear), proteins and lipids [10] through covalent bonds, causing DNA synthesis interruption, leading to the death of *T. cruzi*. In addition, BNZ stimulates phagocytosis in macrophages, contributing to considerable parasitic elimination [11]. Cutaneous manifestations, lymphadenopathy, bleeding, thrombocytopenic purpura, and agranulocytosis are among the most serious adverse reactions of BNZ administration [11]. However, regarding the more specific effects of BNZ on the human salivary gland system, no previous study has specifically investigated the possible harmful effects of the drug on salivary glands [12].

The salivary glands are divided into major (Parotid, Submandibular and Sublingual) and minor salivary glands, which are fundamental structures for oral health and the health of the entire organism. The fluid secreted by salivary glands are provides hydration and lubrication for the surface lining the oral cavity, promotes digestion, and contributes to protection against microorganisms [13–15]. Therefore, the parotid glands produce 50% of salivary volume and is the most important salivary gland in terms of salivary volume production [15], while the submandibular glands have serous content proportional to the mucous content in concentration to the total volume. Thus, fully functional parotid and submandibular glands secrete more than half of the total volume of saliva produced. Given that numerous factors can negatively influence their structure and functionality, maintaining the functionality of such structures is fundamental.

Oxidative imbalance is one of the main factors that can cause glandular dysfunction, which can decrease salivary volume and alter its biochemistry, negatively affecting the patient’s oral health, such as changes in pH that facilitate the appearance of cavities, so it is important to emphasize that as BNZ acts by affecting the redox imbalance in the parasite and in the human body, it cannot be ruled out that it could also cause functional changes in the salivary gland [16]. Moreover, no current definitive therapeutic approach is aimed at mitigating negative symptoms related to the impairment of this tissue and cannot compensate for the damage caused to them [14].

When evaluating Chagas disease, BNZ and salivary glands, the main focus is on the salivary glands of the vector [17], but it has also been demonstrated the effect of BNZ medication on other glands, such as the mammary glands [18] and liver [19]. Other studies have already demonstrated that infection by *Trypanosoma cruzi* affects the parotid gland of rats, through alteration of the epidermal growth factor receptor/testosterone axis, leading to changes in both the acini and the stroma [20,21]. Due to the nature of the mechanism of action of BNZ, it is important to evaluate whether it could also cause changes in this gland.

Furthermore, the toxic effects of BNZ have been widely studied, with deleterious effects already observed in the adrenal, colon and esophagus, being related to its mechanism of action capable of generating reactive metabolites, which bind to proteins, lipids and DNA causing their destabilization, in addition to causing increased production of reactive oxygen species, causing oxidative imbalance [22].

Thus, considering the need for constant use of BNZ in numerous patients and the importance of the salivary glands for health, body homeostasis, and quality of life, this study aimed to evaluate for the first time the possible detrimental effects on salivary biochemistry and salivary gland tissue, as well as to verify possible detrimental changes to parotid and submandibular salivary gland tissue in rats after BNZ administration.

## 2. Materials and methods

### 2.1. Animal experimentation

The experimental procedures were approved by the Ethics Committee for Experimental Animals of the Federal University of Pará (CEUA–UFPA N°: 3133281122) and followed the recommendations of the NIH Guide for the Care and Use of Laboratory Animals and Animal Research: Reporting of *In Vivo* Experiments (ARRIVE) guidelines [23].

**Fig 1:**
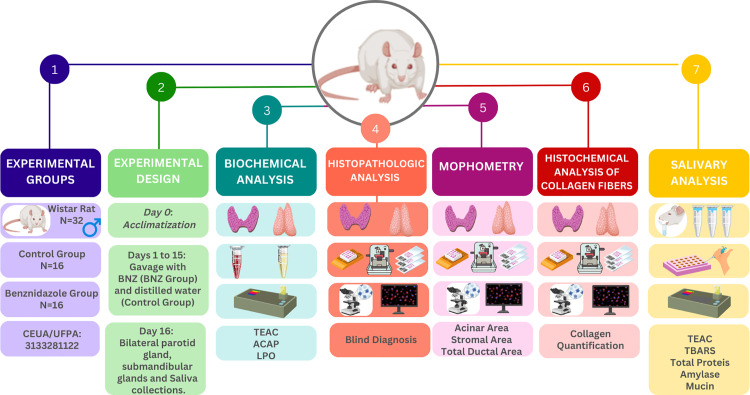
Methodological summary of exposure to benznidazole, collection of glands, and salivary analyses (1) Experimental group allocation; (2) Local experimental design; (3) Biochemical analyses; (4) Morphometry; (5) Picrosirius; (6) Histopathologic Analyses; (7) Salivary analyses.

This study was conducted on 32 male Wistar rats (*Rattus norvegicus*), 66-days-old, weighing approximately 250 g, kept under controlled temperature conditions ranging from 24 ±  2ºC, air humidity between 40 and 70%, and a 12 h lighting cycle between light and dark. All rats were fed standard industrialized rodent feed (Nuvital) and water *ad libitum*. These rats were obtained from the Central Animal Facility of the UFPA, acclimatized to the Experimental Vivarium at the Institute of Health Sciences, and divided into two experimental groups (Fig 1).

The rats were randomly divided into two groups: the control group (n = 16), animals kept under standard laboratory conditions and fed distilled water and BNZ group (n = 16), animals subjected to gavage with BNZ for 15 consecutive days.

### 2.2. Administration of benznidazole

An interspecific allometric extrapolation calculation of the dose was performed to determine the dose of BNZ administered to each animal, based on the therapeutic dose of benznidazole (5 mg/kg) [6] in humans, respecting the corresponding calculation time relationship [24]. This calculation considered a dose of 19.6 mg/kg of BNZ administered once a day for 15 days via intragastric gavage.

### 2.3. Gland collection procedures

On the 16th day, the animals were anesthetized with a mixture of ketamine hydrochloride (90 mg/kg) and xylazine hydrochloride (9 mg/kg). After verifying the absence of corneal reflexes, 16 animals (8 from each group) were injected with pilocarpine (1 mg/kg, Pilocarpine hydrochloride, Sigma-Aldrich, USA) intraperitoneally to enable saliva collection in plastic microtubes on ice for 10 minutes, and the samples were immediately stored at -80 ºC after sample collection. Subsequently, the animals were euthanized by exsanguination, and the major salivary glands were collected and stored while still fresh for analyzing biochemical parameters.

The remaining animals (n =  16) were perfused with 0.9% heparinized saline solution after the same anesthetic process, followed by 4% formaldehyde to collect the parotid and submandibular salivary glands, which were stored in 10% neutral formalin solution for 24 h for histological and histochemical analysis.

### 2.4. Analyses of biochemical parameters of oxidative stress

The parotid and submandibular glands were stored in conic tubes and then freezed with liquid nitrogen and stored together with the saliva samples at -80˚C. The glandular samples were thawed and resuspended in 20 mM Tris-HCl, pH 7.4, at 4˚C, by sonic disaggregation (approximate concentration of 1 g/mL) for analysis. Proteins in the samples were quantified to correct the biochemical data using the method by Bradford et al. [25]. The results are expressed as a percentage of the control.

### 2.5. Determination of total antioxidant capacity equivalent to Trolox (TEAC)

The technique developed by Miller et al. [26] and modified by Re et al. [27] was used to analyze the total antioxidant capacity equivalent to that of Trolox (TEAC/6-hydroxy-2,5,7,8-tetramethylchromono-2-carboxylic acid; Sigma Aldrich 23881-3, USA), a water-soluble antioxidant analogous to vitamin E. The colorimetric method is based on the reaction between ABTS (Sigma Aldrich A1888, USA) and potassium persulfate (K_2_S_2_O_8_; Sigma-Aldrich 60490) that react and results in an ABTS+• radical (2,2-azinobis radical [3-ethylbenzothiazoline-6-sulfonate], diammonium salt), which is a coloring chromophore with green and blue hues. The results were expressed as µM/g of protein in the hemolyzed tissue [28].

### 2.6. Antioxidant capacity test against peroxyl radicals (ACAP)

ACAP was analyzed by quantifying the reactive oxygen species (ROS) produced in each equally concentrated sample (2.5 µg of protein/µL) after exposure to a peroxyl radical generator [29]. Thermal decomposition (35◦C) of 2,2’-azobis 2-methylpropionamidine dihydrochloride (ABAP; 4 mM; Sigma-Aldrich, USA) produced the peroxyl radicals. The compound 2’,7’-dichlorofluorescein diacetate (H2DCF-DA, Invitrogen™, Whaltan, MA, USA) was used at a final concentration of 40 nM. The relative difference between the ROS areas with and without ABAP was considered to measure the antioxidant capacity. The results are expressed as the inverse of the relative area.

### 2.7. Lipid peroxidation assay (LPO)

LPO levels were analyzed by measuring malondialdehyde (MDA) levels [30]. The lysates were centrifuged at 4000 ×  g for 10 min at 4ºC. Then, the supernatants and standard MDA solutions were incubated together with a solution of 10.3 mM methanesulfonic acid and N-methyl-2-phenylindole diluted in methanol at 45 ºC for 40 min, followed by spectrophotometric reading (λ =  570 nm). The results were expressed in nanomoles per microgram (nM/µg) of protein and represented as a percentage of the control.

### 2.8. Histological analyses

After fixing the samples in formaldehyde, the glands of each animal were postfixed in 4% formaldehyde until processing. They were then dehydrated with increasing concentrations of ethanol (70%, 80%, 90%, absolute 1, and absolute 2), diaphanized in xylol, and embedded in paraffin. Sections of 7 µm thick were obtained using a manual microtome, which were then stained with hematoxylin and eosin. For quantitative analysis, photomicrographs of five cross-sections of the glands were obtained using a digital color camera (Cyber-Shot DSC W-230, Sony, Tokyo, Japan) coupled with a microscope (Eclipse E200, Nikon, Tokyo, Japan; 40 × magnification), and three fields of each section were evaluated.

### 2.9. Histopathological analysis

Two blinded pathologists independently analyzed the slides qualitatively using a microscope (Eclipse E200, Nikon, Tokyo, Japan; magnification, × 40). Cohen’s kappa statistic was applied to assess interobserver agreement, revealing excellent agreement (κ =  0.93). Secretory units, ductal system, and surrounding stroma were examined to identify possible pathological changes in glandular tissue, lining epithelium, and connective tissue. Representative photomicrographs were obtained using a color digital camera (Cyber-Shot DSC W-230; Sony, Tokyo, Japan) attached to a microscope (Eclipse E200; Nikon, Tokyo, Japan; magnification, × 40).

### 2.10. Morphometric analysis

The tissue morphometric evaluation variables were expressed in µm² were total area of stroma, acinus, and total ductus [31–33]. A digital image analyzer (ImageJ software, v. 1.53; NIMH of Health, Bethesda, MD, USA; http://rsbweb.nih.gov/ij/) obtained the variable values.

### 2.11. Histochemical analysis of collagen fibers

The samples were stained with Picrosirius Red [34] and three sections of each were examined under a polarized light microscope (Eclipse E200, Nikon, Tokyo, Japan; 40 × magnification), and three photomicrographs of each region were obtained using a digital color camera (Cyber-Shot DSC W-230, Sony, Tokyo, Japan) attached to the microscope to assess the collagen content of the salivary glands. The threshold tool in the ImageJ software was used to delineate the collagen area, using the arithmetic mean of the sections to determine the collagen area of the sample, which was measured in um^2^.

### 2.12. Salivary analysis

#### 2.12.1. Total protein.

Total protein levels were determined according to the protocol proposed by Bradford [25] in which proteins were bound to Coomassie brilliant blue dye to form a blue compound with a maximum absorbance at 595 nm using a spectrophotometer. The obtained data were tabulated and compared with the reference values stipulated by Giacomello et al [35]. The results were expressed as g/dL and presented as a percentage of the control.

#### 2.12.2. Amylase activity.

The amylase activity was determined according to the method described by Caraway [36] and modified by Rinderknecht [37] and Searcy [38], in which colorimetric detection was performed (K003 BIOCLIN, Quibasa, Belo Horizonte, Minas Gerais, Brazil). Absorbance was measured spectrophotometrically at 660 nm. The results were expressed as U/dL and represented as a percentage of the control.

#### 2.12.3. Mucin.

Mucin corresponds to a type of glycoprotein with high molecular weight, with viscoelastic properties that have lubricating capacity, favors the hydration of oral surfaces due to its high degree of glycosylation, avoiding possible desiccation [39], makes up the acquired dentin film and is related to dental protection [40]. Mucin was analyzed using a kit to detect protein concentration (Bioclin, Belo Horizonte, Minas Gerais, Brazil). The results were expressed as mg/dL and presented as a percentage of the control.

### 2.13. Statistical analyses

Data were statistically analyzed using GraphPad Prism (version 8.0; San Diego, CA, USA). The Shapiro–Wilk test was used to verify the normality of the data. The student’s t-test for parametric data was used for normally distributed variables, assuming a statistical significance value of p < 0.05. Results are expressed as mean ±  standard error.

## 3. Results

### 3.1. BNZ caused oxidative imbalance in salivary glands

**Fig 2:**
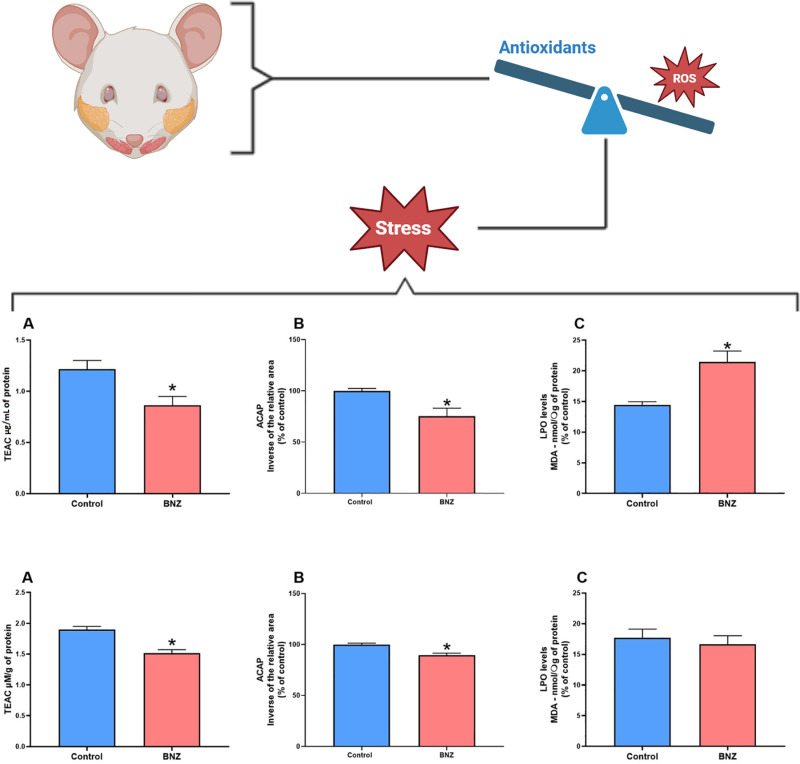
Effects of exposure to benznidazole (19.6 mg/kg mg/kg/day) for 15 days on the oxidative biochemistry of the salivary glands. Parotid gland: (A) Determination of total antioxidant capacity equivalent to Trolox (TEAC). (B) Antioxidant capacity against peroxyl radicals (ACAP). (C) Lipid Peroxidation Assay (LPO). Submandibular gland: (D) Determination of total antioxidant capacity equivalent to Trolox (TEAC). (E) Antioxidant capacity against peroxyl radicals (ACAP). (F) Lipid Peroxidation Assay (LPO). Results are expressed as the mean ±  standard error of the mean (SEM) in % of control. Student’s t test significance (*p <  0.05).

The assessment of oxidative biochemical pathways in the parotid gland showed a reduction in TEAC antioxidant capacities and ACAP in the BZN exposed group (0.8625 ±  0.08683 μg/mL; p =  0.0259 and 75.38 ±  3.153; p < 0.0001, respectively) compared with the controls (1.218 ±  0.08390 μg/mL and 100.0 ±  0.9964, respectively), with a statistically significant difference (Figs 2A and 2B). The exposure to BNZ caused an increase in lipid peroxidation in the parotid gland in the exposed group (21.44 ±  1.769 nM/µg; p = 0.0053) compared with the control group (14.46 ±  0.4928 nM/µg; p = 0.0053), with a significant difference (Fig 2).

Regarding the submandibular gland, a significant reduction in the ACAP antioxidant pathway was observed in the exposed group (89.57 ±  0.7792; p < 0.0001) compared with the control (100.0 ±  0.5208). In relation to TEAC, the BNZ group showed reduction (1.516 ±  0.122 μg/mL; p = 0.243) compared with the control group (1.692 ±  0.287 μg/mL), while in the LPO pro-oxidant pathway, no significant difference was observed between the group exposed to BNZ (1.516 ±  0.122 nM/µg; p = 0.243) and the control (1.692 ±  0.287 nM/µg).

### 3.2. Administration of BNZ caused the appearance of areas of inflammation in the salivary gland

**Fig 3:**
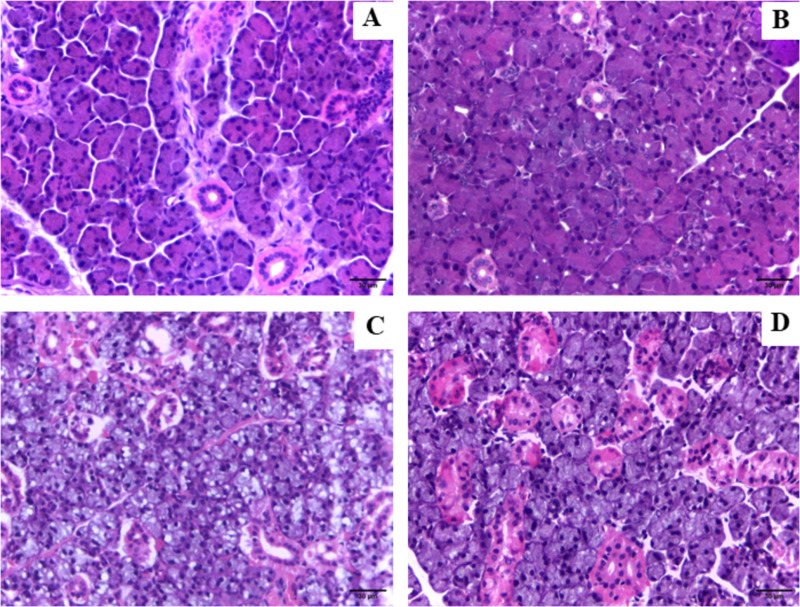
Effects of benznidazole exposure on rat salivary glands. Photomicrograph representation in (A) parotid control, (B) parotid BNZ, (C) submadibular control and (D) submandibular BNZ.

Based on the findings in the parotid gland, it was observed that, in the control group, normal glandular parenchyma was found (Fig 3A), while a mononuclear inflammatory infiltrate was observed dispersed throughout the stroma with the presence of a focal area of lymphocytes and mast cells in the group treated with BNZ (Fig. 3B). In the submandibular gland, the control group also presented normal glandular parenchyma (Fig 3C; in contrast, the BNZ group revealed few lymphocytes and mast cells in the stroma and an exuberant ductal system (Fig 3D).

### 3.3. BNZ increased in the area of acini and a decrease in the area of stroma in salivary glands

**Fig 4:**
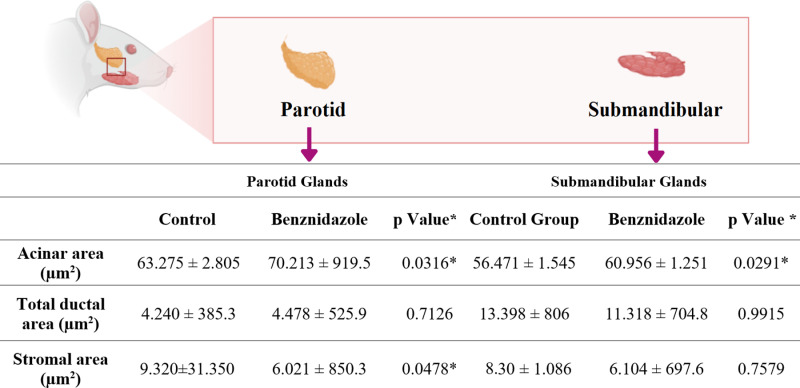
Morphometric measurements of rat salivary glands after exposure to benznidazole n =  16/group (mean ±  SEM). *  (Student’s t test, p <  0.05).

Morphometric analysis demonstrated that compared with the control group, the group exposed to BNZ may be associated with the parotid gland, a reduction in the stromal area, and an increase in the average area of the acini, with a statistically significant difference (Fig 4). However, when the average duct area was analyzed, no significant difference was observed between the exposed and control groups.

### 3.4. BNZ decreased the area of collagen in the parotid gland and an increase in the submandibular gland

**Fig 5:**
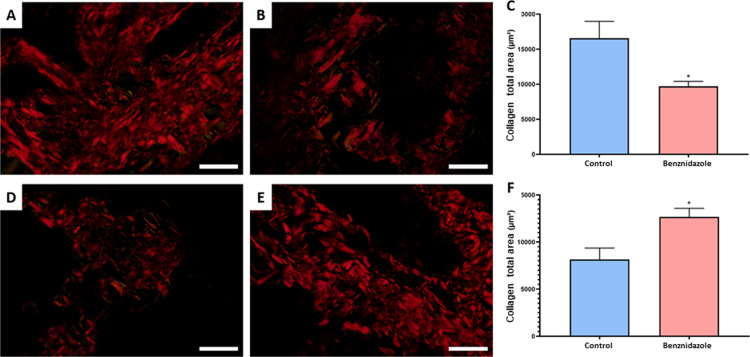
Representative photomicrographs of the analysis of collagen areas in the parotid and submandibular salivary glands of rats administered BNZ (19.6 mg/kg) for 15 days. A represents the parotid gland of animals in the control group, B the parotid gland of the BNZ group, D the submandibular gland of the control group and E the submandibular gland of the BNZ group. C shows the graph of the collagen area analysis for the parotid gland comparing the control group and the BNZ group and F shows the graph of the collagen area analysis for the submandibular gland comparing the control group and the BNZ group. Results were evaluated in µm, expressed as mean +  standard error of the mean, using the Shapiro Wilk normality test and for comparison purposes, the Student’s T test was used, the significance value adopted was p < 0.05.

The parotid gland evaluation made it possible to observe a statistically significant reduction in the collagen area in the BNZ group (9.707 ±  692.3 µm²) compared with the control group (16.577 ±  2.396 µm²; p = 0.0079). However, the result in the submandibular gland was a significant increase in collagen area in the BNZ group (12.673 ±  9.108 µm²), to the detriment of the control group (8.148 ±  1.218 µm²; p = 0.0248); both results and representative photomicrographs are shown in fig 5.

### 3.5. BNZ caused oxidative imbalance and functional damage in saliva

**Fig 6:**
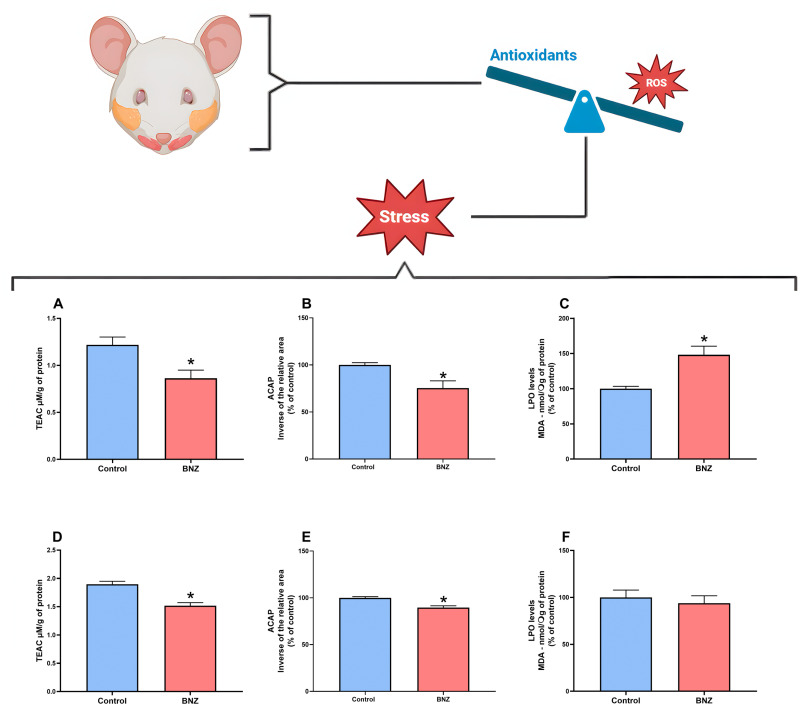
Biochemical parameters and redox state markers.in saliva. (A) Determination of total antioxidant capacity equivalent to Trolox (TEAC). (B) Determination of levels of substances reactive to thiobarbituric acid (TBARS). (C) Determination of total protein. (D) Determination of amylase. (E) Determination of mucin. Results are expressed as the mean ±  standard error of the mean (SEM) in % of control. Student’s t test significance (*p < 0.05).

When checking the oxidative analysis of the animals’ saliva, it was observed that the TEAC antioxidant capacity in the group exposed to BNZ (73.08 ±  4.419 μg/mL; p = 0.0154) showed a reduction in salivary antioxidant capacity compared with the control group (100.0 ±  6.706 μg/mL), with a statistically significant difference (Fig 6A). Contrarily, compared with the control group (100.0 ±  4.499 µ M/mL), the group exposed to BNZ showed an increase with a statistically significant difference in the TBARS pro-oxidant pathway (167.6 ±  3.225 µ M/mL; p = 0.4827) (Fig 6B).

Regarding the determination of total proteins in saliva, the exposed group (84.26 ±  0.8723 g/dL; p = 0.0006) showed a reduction in the levels of total proteins compared with the control group (100.0 ±  2.258 g/dL) (Fig 6C). On analysis of the deposition of salivary amylase, the exposed group demonstrated a reduction of this protein with a statistical difference (72.48 ±  2.173 g/dL; p < 0.0001) compared with the control group (100.0 ±  0.9151 g/dL) (Fig 6D). For biochemical analysis of mucin, a reduction in mucin protein was also demonstrated in the exposed group (77.45 ±  2.104 g/dL; p = 0.0226), when compared with the control (100.0 ±  7.098 g/dL), with a statistically significant difference (Fig 6E).

## 4. Discussion

The results of the present study demonstrate for the first time that BNZ administration is associated with salivary gland disfunction, induced by impairment of the redox state by BNZ, characterized by increased lipid peroxidation and reduced antioxidant capacity in both salivary glands. It is important to demonstrate the possible effects of this drug on the salivary glands and saliva because BNZ, to this day still the best treatment for Chagas’ disease, and because of its long duration in affecting the quality of saliva, could cause the emergence of new oral diseases.

Morphological aspects such as an increase in the acinar area in both glands and a reduction in the stromal area in the parotid gland, are also accompanied by a reduction in the area of collagen fibers. Furthermore, the presence of focal areas of inflammation with lymphocytes in both glands and focal areas of inflammation in the exposed group were observed from the histopathological analysis. Additionally, the effects of BNZ on the salivary glands had a negative impact on the salivary redox state, as evidenced by the increase in lipid oxidative damage and reduction in antioxidant capacity, and also on the biochemical composition of saliva, as characterized by the prejudice in the secretion of total protein, mucin and amylase.

Therefore, the exposure model adopted in this study was applied to simulate the ingestion of BNZ in humans at a concentration of 5 mg/kg, corresponding to a dosage of 19.6 mg/kg in rats, and was administered for 15 days daily [24]. Other studies in animal models have analyzed the possible effects of BNZ on other systems, such as mammary glands [18], ovaries [41], testicles [42], liver [43] and colon [44]; however, no studies have investigated the possible effects of BNZ on salivary glands. Therefore, the clinical protocol adopted by the National Commission for the Incorporation of Technologies in the SUS (Brazil) and the therapeutic guidelines for Chagas Disease were used and reproduced in rats after allometric calculation [10].

The redox system in organisms is an adaptive biochemical system in response to chemical, physical, or biological stimuli, in which there is an exchange of electrons between the oxidative species. Thus, the redox system corresponds to a specialized mechanism for controlling oxidative stress, which is balanced by the production of free radicals, a physiological process arising from metabolic reactions in homeostasis. However, oxidative damage resulting from excess production [45] requires mediation by the activation of the antioxidant defense mechanism [46], which intercept free radicals, preventing the loss of cellular integrity and lesion formation [47]. Therefore, oxidative stress caused by the generation of excess free radicals or a reduction in the speed of their removal by antioxidants leads to biomolecular oxidation that triggers an evident homeostasis imbalance and biological functional loss.

When analyzing the effects of BNZ on other murine glands, similar patterns were identified: in the colon, damage occurred due to the interaction of BNZ metabolites with mucosal proteins during their activation by nitroreductases, in addition to greater production of free radicals [44]; in the ovaries, due to the presence of nitroreductases in the mitochondria, the greatest damage occurs in this organelle, due to the redox imbalance caused by the greater amount of ROS generated [41]; in the testes, no immediate damage was seen, but the BNZ metabolites were covalently bound, which can lead to the appearance of tissue damage, especially at longer treatment times [42].

Owing to the potential oxidative damage to cells and tissues [48], this process was also observed in the results of biochemical analyses of the glands. In this context, it is possible to identify patterns of oxidative damage in the parotid gland, as benznidazole decreased the levels of antioxidant markers (TEAC and ACAP), and increased the levels of prooxidant markers (LPO). This profile signals that BNZ directly affects the oxidative balance of the parotid gland. A similar process occurs in the submandibular gland, with only LPO levels showing no significant differences, which may indicate that oxidative damage takes longer to occur in this gland or does not occur at all. The decrease in TEAC and ACAP levels shows that benznidazole caused oxidative damage and, as a way of dealing with this damage, the body used natural antioxidants to sequester these radicals and reduce cell damage, which is why there are lower levels in the BNZ group, as they were used against, demonstrating the body’s attempt to recover oxidative homeostasis [46].

The oxidative changes observed in this study may be related to the mechanism of action of BNZ, which occurs because of the reduction of the prodrug within the cell, contributing to the formation of free radical intermediates and nucleophilic metabolites [46], it also causes oxidative deregulation both in *Trypanosoma cruzi* and in the host cells, and as demonstrated in this study, it was able to affect the major salivary glands. Being the molecular structure of this pro-drug composed of nitrogen, oxygen, carbon, and hydrogen, when administered, the reaction begins through nucleophilic metabolite intermediates, where the action of nitroreductors that act specifically on RNO_2_ [49] molecular systems reduce the nitro group (NO_2_) into an amino group (NH_2_). This reaction generates a nitro radical that can bind to the fundamental macromolecules of the parasite as well as the host, such as DNA (both mitochondrial and nuclear), proteins, and lipids through covalent bonds.

Furthermore, it is worth mentioning that BNZ also increases phagocytosis as it increases the available amount of the cytokine interferon gamma, causing cell lysis [49] and proven to induce oxidative stress [22]. Therefore, the release of free radicals during their action can cause oxidative damage to glandular tissues and is associated with inflammation due to the release of inflammatory cytokines secreted in cases of oxidative stress.

Thus, the presence of ROS, stimulated by the oxidative stress process found in our analyses, is in agreement with the histopathological findings that indicated the presence of lymphocytes in glands, mast cells and focal areas of inflammation. The appearance of such cells and areas of inflammation indicates a glandular inflammatory process because analysis found that at the injury site during inflammatory processes, epithelial and endothelial cells release inflammation cascade initiating factors, attracting defense cells to the region, such as lymphocytes and mast cells [50] found during the analysis. This condition indicates the beginning of salivary gland inflammation, which can cause symptoms, such as increased temperature in the region and stiffening of the inflamed glandular area [51].

Therefore, the biochemical and histopathological findings demonstrated the presence of oxidative and inflammatory stress, which may be associated with structural changes [52] found in morphometric analyses, such as an increase in the acinar area in both glands and a reduction in the stromal area in the parotid gland.

Furthermore, histochemical analysis showed a reduction in the area of collagen fibers in the parotid gland, accompanied by a reduction in the gland’s stroma. Since oxidative stress is capable of modulating collagen synthesis [53], the result does not follow the submandibular gland, which showed an increase in the collagen area of the exposed group; however, even though they perform the same function, the glands have different cellular metabolisms, with the submandibular gland being mainly anaerobic [54] and apparently less susceptible to damage from oxidative stress [55,56]^.^

In addition, the literature still points to inflammatory cases in that when there is a loss of stromal cells, compensation occurs through a collagen increase [57]. Although no major changes were observed, it was assumed that the exposure time was insufficient to detect more serious morphometric damage. However, it is speculated that the increase in the acini area may indicate a compensatory reaction of the glandular tissue faced with inflammation, in which the tissue seeks to increase in volume to compensate for structural damage that could impair its function, which in this case is the secretion of salivary fluid. The stromal area reduction in the parotid may be due to connective tissue compression caused by the increase in the area of the acini. These morphological and functional changes may be related to the anticholinergic effect of BNZ by modulating the activity of muscarinic receptors and interferon-λ, which can interfere with saliva secretion as well as the production of saliva proteins [58].

This theory corresponds to the findings of the salivary analyses, in which a decrease in antioxidant activity (decrease in TEAC levels) and an increase in pro-oxidant levels (increase in TBARS levels) were observed in the group exposed to BNZ, indicating oxidative stress in the salivary fluid [48]. Regarding the analysis of the functional quality of saliva, there was a significant reduction in total proteins in the BNZ group, and the activity of amylase and mucin was also reduced, suggesting possible alterations in salivary functions associated with the biochemical alterations found in the exposed group.

Amylase is an important organic component of saliva, being an enzyme involved in digestive processes and favoring bioadhesion in humans [59] participating in the binding of oral bacteria [60]. This may represent possible injuries to the saliva excretory tissue, causing harm to health, salivary quality, and quality of life, as these enzymes are responsible for digestive processes [61] protection, and lubrication of epithelial surfaces [8,62] and can promote swallowing difficulties, dryness of the oral mucosa, and dry mouth sensation.

Thus, based on the biochemical, morphological, and functional changes found, it is possible to verify that BNZ was capable of causing changes in the tissue structure of the submandibular and parotid glands when comparing the exposed group with the control group, which negatively impacts the proper functioning of these glands and reduces the effectiveness of their secretory product, saliva. It also compromises the oral and systemic homeostasis of patients, given the fundamental role of the salivary glands and salivary fluid in the body as a whole, both in terms of health and quality of life.

The excretion of BZN in saliva is not scientifically proven. However, the findings of this study demonstrate that in its distribution phase throughout biological compartments, this medication may cause oxidative damage in the salivary glands [63], leading to inflammatory damage, compromising the proper functioning of this system.

Summarily, the findings of this study suggest that the ingestion of BNZ induces glandular oxidative stress due to an increase in pro-oxidant levels and a decrease in antioxidants in the glands and saliva, with strong evidence of inflammation related to this biochemical imbalance. This was evidenced by the sites of inflammation and inflammatory cells present in the tissue and decreased functional quality of excreted saliva, with a direct impact on the reduction of mucin levels in the group exposed to the prodrug. Although this was not the focus of this study, it is interesting that other studies have been carried out to verify the potential effects of BNZ on salivary glands over a longer period of drug exposure, as well as to carry out analyses that evaluate the buffering capacity of saliva and salivary pH.

Although our work provides new paradigms for the toxicity of BNZ in salivary glands, it is important to emphasize that translational studies in humans should be carried out in the future to see if these effects also occur in humans. Furthermore, the analyses were carried out with stimulated saliva, and it is very important to check saliva at different times of the day to ensure greater accuracy of these studies in humans.

## 5. Conclusions

Through this preclinical study, we observed that the administration of BNZ promoted inflammation caused by oxidative stress associated with morphological damage and imbalance in the salivary glands and biochemical and functional changes in saliva, highlighting the potential damage caused by the drug.

## Supporting information

S1 Table Parametric results of the biochemical analysis of the levels of determination of total antioxidant capacity equivalent to Trolox (TEAC), antioxidant capacity against peroxyl (ACAP) and lipid peroxidation (LPO) levels. (DOCX)
