# Supplementary material for: Investigation of the effects of benznidazole on the salivary glands: A biochemical, morphological, and functional approach
Source: PLoS One. 2025 Jun 3;20(6):e0317876. doi: 10.1371/journal.pone.0317876 (PMC12132995; doi:10.1371/journal.pone.0317876)
Supplement: S1 Table — (DOCX) [file pone.0317876.s001.docx]

**Investigation of the effects of Benznidazole on salivary gland: A biochemical, morphological and functional approach Benznidazole effect in salivary gland**

Emanuelly Camilly Soares de Lima-da-Silva^1^, Wallacy Watson Pereira Melo^1^, Paulo Fernando Santos Mendes^1^, Cristian dos Santos Pereira^1^, José Mario Matos-Sousa^1^, Cristian Kallahan Silva Chagas^1^, Hannah Gil de Farias Morais^2^, Roseana de Almeida Freitas^2^, Antonio Hernandes Chaves-Neto^4^, Rafael Rodrigues Lima^1^*

1. Functional and Structural Biology, Institute of Biological Sciences, Federal University of Pará, Belém, Pará, Brazil

2. Department of Oral Pathology, Federal University of Rio Grande do Norte, Brazil

3. Department of Basic Sciences, School of Dentistry of Araçatuba, Universidade Estadual Paulista, Araçatuba, São Paulo, Brazil.

4. Department of Basic Sciences, School of Dentistry of Araçatuba, Universidade Estadual Paulista, Araçatuba, São Paulo, Brazil

*Corresponding author:

Rafael Rodrigues Lima, Ph.D. Laboratory of Functional and Structural Biology, Institute of Biological Sciences. Federal University of Pará, 01 Augusto Corrêa Street, Guamá. ZIP CODE 66075- 110. Belém, Pará, Brazil.

E-ail: rafalima@ufpa.br

| Supplementary table I. Parametric results of the biochemical analysis of the levels of determination of total antioxidant capacity equivalent to Trolox (TEAC), antioxidant capacity against peroxyl (ACAP) and lipid peroxidation (LPO) levels. In addition, the results of the morphometric analyses were total stromal area, total acinar area, total duct area and histochemical analysis of collagen fibers (µm^2^) of the parotid and submandibular glands. Finally, there are the salivary analyses of TEAC, Determination of levels of substances reactive to thiobarbituric acid (TBARS), Total Proteins, Amylase and Mucin of rats exposed to benznidazole. Results are expressed as the mean ± standard error of the mean (SEM). Student's t test significance (*p < 0.05). | | | |
| --- | --- | --- | --- |
|  | **Control** | **BNZ** | **P value** |
| Parotid gland |  |  |  |
| TEAC | 1.218 ± 0.08390 | 0.8625 ± 0.08683* | 0.0259 |
| ACAP | 100.0 ± 0.9964 | 75.38 ± 3.153* | <0.0001 |
| LPO | 14.46 ± 0.4928 | 21.44 ± 1.769* | 0.0053 |
| Submandibular gland |  |  |  |
| TEAC | 1.897 ± 0.05207 | 1.516 ± 0.1222 | 0.0357 |
| ACAP | 100.0 ± 0.5208 | 89.57 ± 0.7792* | <0.0001 |
| LPO | 17.73 ± 1.391 | 16.63 ± 1.411 | 0.6005 |
| Morphometric and histochemical analyses | | | |
|  | **Control** | **BNZ** | **P value** |
| Parotid gland |  |  |  |
| Stromal área (µm^2^)  Acinar área (µm^2^)  Total ductal area (µm^2^) | 9.320 ± 31.350  63.275 ± 2.805  4.240 ± 385.3 | 6.021 ± 850.3*  70.213 ± 919.5*  4.478 ± 525.9 | 0.0478  0.0316  0.7126 |
| Collagen area | 16.577 ± 2.396 | 9.707 ± 692.3* | 0.0079 |
| Submandibular gland |  |  |  |
| Stromal área (µm^2^) | 8.30 ± 1.086 | 6.104 ± 697.6 | 0.7579 |
| Acinar área (µm^2^) | 56.471 ± 1.545 | 60.956 ± 1.251* | 0.0291 |
| Total ductal area (µm^2^) | 13.398 ± 806 | 11.318 ± 704.8 | 0.9915 |
| Collagen area (µm^2^) | 8.148 ± 1.218 | 12.673 ± 9.108* | 0.0248 |
| Saliva | **Control** | **BNZ** | **P value** |
| TEAC | 100.0 ± 6.706 | 73.08 ± 4.419* | 0.0154 |
| TBARS | 100.0 ± 4.499 | 167.6 ± 3.225* | 0.4827 |
| Total Proteins | 100.0 ± 2.258 | 84.26 ± 0.8723* | 0.0006 |
| Amylase | 100.0 ± 0.9151 | 72.48 ± 2.173* | <0.0001 |
| Mucin | 100.0 ± 7.098 | 77.45 ± 2.104* | 0.226 |
